# Correlating Structural Properties with Catalytic Stability in Nanocrystalline La(Sr)CoO_3_ Thin Films Grown by Pulsed Electron Deposition (PED)

**DOI:** 10.3390/ma18194550

**Published:** 2025-09-30

**Authors:** Lukasz Cieniek, Dominik Grochala, Tomasz Moskalewicz, Agnieszka Kopia, Kazimierz Kowalski

**Affiliations:** 1 Faculty of Metals Engineering and Industrial Computer Science, AGH University of Krakow, al. Mickiewicza 30, 30-059 Krakow, Poland; tmoskale@agh.edu.pl (T.M.); kopia@agh.edu.pl (A.K.); kazimierz.kowalski@agh.edu.pl (K.K.); 2 Faculty of Computer Science, Electronics and Telecommunications, AGH University of Krakow, al. Mickiewicza 30, 30-059 Krakow, Poland; grochala@agh.edu.pl

**Keywords:** perovskite, thin films, Pulsed Electron Deposition (PED), channel spark discharge, gas sensor, NO_2_, LaCoO_3_, Sr doping

## Abstract

This study investigates the structural, morphological, and gas-sensing properties of pure and strontium-doped lanthanum cobaltite (La_1−x_Sr_x_CoO_3_) perovskite thin films obtained by Pulsed Electron Deposition (PED). This sustainable ablative technique successfully produced high-quality, dense, nanocrystalline films on Si and MgO substrates, demonstrating excellent stoichiometric transfer from the source targets. A comprehensive analysis using XRD, SEM, TEM, AFM, and XPS was conducted to characterize the films. The results show that Sr-doping significantly refines the microstructure, leading to smaller crystallites and a more uniform surface topography. Gas sensing measurements, performed in a temperature range of 100–450 °C, revealed that all films exhibit a characteristic p-type semiconductor response to nitrogen dioxide (NO_2_). The La_0.8_Sr_0.2_CoO_3_ composition, in particular, demonstrated the most promising performance, with enhanced sensitivity and excellent operational stability at temperatures up to 350 °C. These findings validate that PED is a reliable and precise method for fabricating complex oxide films and confirm that Sr-doped LaCoO_3_ is a highly promising material for developing high-temperature NO_2_ gas sensors.

## 1. Introduction

Escalating industrialization has exacerbated air pollution, posing significant threats to both the environment and human health. Consequently, the effective monitoring and control of gaseous pollutants such as CO, NO, NO_2_, NH_3_, and SO_2_ have become critical. Gas sensors are indispensable for this purpose, with vital applications in industrial safety, public air quality monitoring, and emission control systems [1].

A key area of application is the automotive sector, where stringent regulations like the European Union’s Euro standards mandate the reduction in harmful vehicle emissions. Selective Catalytic Reduction (SCR) systems in diesel engines are the primary technology for neutralizing nitrogen oxides (NO_x_) [2]. An SCR system’s efficiency relies critically on a fast and accurate NO_2_ sensor. This sensor provides real-time data to the engine control unit (ECU), which then precisely meters a urea-based solution (AdBlue) into the exhaust stream. This process catalytically converts toxic NO_x_ into harmless nitrogen (N_2_) and water (H_2_O) [3]. Therefore, the sensor’s performance is paramount for maximizing emission reduction while preventing operational issues like ammonia slip [4].

However, conventional sensor technologies face significant limitations. Traditional thick-film sensors often lack the required sensitivity and response time, while some thin-film alternatives can suffer from long-term stability issues [5]. These drawbacks have spurred research into alternative materials. Perovskite-type oxides are particularly promising candidates, offering excellent thermal stability and tunable electrical properties suitable for high-performance gas sensing [6]. Perovskites incorporating transition metals such as manganese (Mn), cobalt (Co), or iron (Fe) are exceptionally promising due to their versatile electronic structures and catalytic activity [7].

A critical aspect of this research is the use of advanced ablative deposition techniques to synthesize thin film sensors. While traditional methods like Chemical Vapor Deposition (CVD) and sputtering are industry standards, they are associated with significant environmental drawbacks. These techniques often rely on hazardous chemical precursors and carrier gases, leading to toxic waste generation. In contrast, ablative techniques, such as Pulsed Electron Deposition (PED), offer a more sustainable and efficient pathway for producing high-quality thin films [8]. As a physical deposition method, PED eliminates the reliance on toxic and volatile chemicals, minimizing hazardous byproduct generation. It also provides exceptional control over film thickness, crystallinity, and microstructure at lower substrate temperatures, leading to improved device performance and reduced energy consumption [9].

A cornerstone of this work is the strategic doping of the perovskites with strontium (Sr). Substituting lanthanum (La^3+^) with strontium (Sr^2+^) in lanthanum cobaltite (LaCoO_3_) induces charge-compensating defects, such as oxygen vacancies and changes in the oxidation state of the B-site cations (Co). These modifications are paramount for enhancing gas sensing performance, as oxygen vacancies create more active sites for gas adsorption and dissociation. This defect engineering approach is crucial for developing highly sensitive and selective resistive sensors [10]. By combining the tailored properties of Sr-doped perovskites with the environmental and practical benefits of ablative fabrication, this research aims to advance the development of next-generation thin-film sensors.

## 2. Research Material

Lanthanum cobaltite (LaCoO_3_) perovskite oxide is a highly promising material for catalytic applications due to its unique combination of electronic, magnetic, and structural properties. A key advantage is its remarkable mixed ionic and electronic conductivity, which is crucial for processes requiring efficient charge transfer and oxygen mobility, such as in solid oxide fuel cell (SOFC) cathodes, oxygen separation membranes, and catalysts for CO oxidation [11].

Structurally, LaCoO_3_ is exceptionally stable, maintaining its rhombohedral (R3c) or orthorhombic (Pbnm) crystal structure up to a high melting point of 1740 °C. The material’s properties can be precisely tailored by inducing structural distortions through temperature changes or cation substitution at the B-site. A defining catalytic characteristic of LaCoO_3_ is the tunable spin state of its cobalt ions, a feature rarely observed in other magnetic oxides [12]. Materials’ electronic behavior is governed by the presence of mixed Co^3+^/Co^4+^ valence states. Below 100 K, Co^3+^ ions adopt a low-spin state. With increasing temperature, they undergo a gradual transition to intermediate- and high-spin states. This spin-state transition, which can also be influenced by external gas pressure, directly modulates the electronic structure by altering interatomic distances and bond angles, thereby influencing the material’s magnetic and catalytic activity [13,14].

The conductivity mechanism in LaCoO_3_ is highly temperature-dependent. Between 110 K and 350 K, its semiconducting behavior is governed by a small-polaron hopping mechanism. Above 650 K, the material undergoes a semiconductor-to-metal transition, leading to a significant increase in p-type electronic conductivity. This transition is associated with the stabilization of high-spin cobalt ions, which creates a partially filled electron shell that facilitates excellent charge transport [15].

Crucially for catalysis, the material’s ionic conductivity—driven by the diffusion of oxygen ions through lattice vacancies—is also enhanced at elevated temperatures. This synergy between high electronic and ionic conductivity is a major advantage for catalytic processes that depend on both electron transfer and oxygen activation. This unique combination of high thermal stability, tunable electronic structure, and excellent mixed conductivity makes LaCoO_3_ a versatile and highly effective catalyst, with demonstrated potential in applications ranging from SOFCs to low-temperature CO detection [16].

Building upon the preceding discussion of their gas-sensing properties, this study focuses on the fabrication and comprehensive analysis of La(Sr)CoO_3_ perovskite thin films, examining their structure and catalytic activity. The selection of a suitable deposition technique is paramount for producing high-quality functional films. Pulsed Electron Deposition (PED) presents a highly advantageous solution, primarily due to its unique capability for stoichiometric transfer. This process effectively preserves the phase and chemical composition of the target material in the resulting nanocrystalline film, crucially avoiding gas-phase decomposition. Nevertheless, successful deposition is contingent upon the meticulous selection of the substrate and the precise optimization of key process parameters. This paper elucidates these critical factors, providing a methodological framework for depositing high-quality perovskite thin films via the PED technique.

## 3. Methodology and Research Techniques

### 3.1. Target Fabrication for PED Process

This study’s initial phase focused on the fabrication of perovskite targets for subsequent Pulsed Electron Deposition (PED). The targets were derived from nanopowders synthesized through a mechanical alloying route using high-purity La_2_O_3_, Co_3_O_4_, and SrO as base materials. The starting materials for target fabrication via mechanical synthesis were sourced from a reputable global supplier (Kurt J. Lesker, Sigma-Aldrich/Merck, St. Louis, MO, USA). The quality of all powders was verified before use. As per the manufacturer’s specifications, the materials met the required purity and particle size: Lanthanum(III) oxide (La_2_O_3_, 99.99% trace metals basis), Cobalt(II,III) oxide (Co_3_O_4_, nanopowder, <50 nm, 99.5% trace metals basis), and Strontium oxide (SrO, 99.9% trace metals basis). Prior to synthesis, these powders were calcined to remove residual moisture, thereby ensuring the formation of the correct perovskite phase with the intended stoichiometry. The high-energy milling process was carried out in a planetary ball mill Retsch PM 100 (Retsch GmbH, Retsch-Allee 1-5, 42781 Haan, Germany) for a total duration of 28 to 30 h at a rotational speed of 550 to 650 RPM, employing cycles of 2-h milling and 30-min rests. Three distinct perovskite materials were successfully synthesized: stoichiometric LaCoO_3_, alongside two Sr-doped compositions, La_0.9_Sr_0.1_CoO_3_ and La_0.8_Sr_0.2_CoO_3_. The procedure for preparing targets for the electron ablation process is identical to that for preparing materials for the laser ablation process mentioned in the previous summary [17].

### 3.2. Perovskite Thin Film Deposition

**Figure 1:**
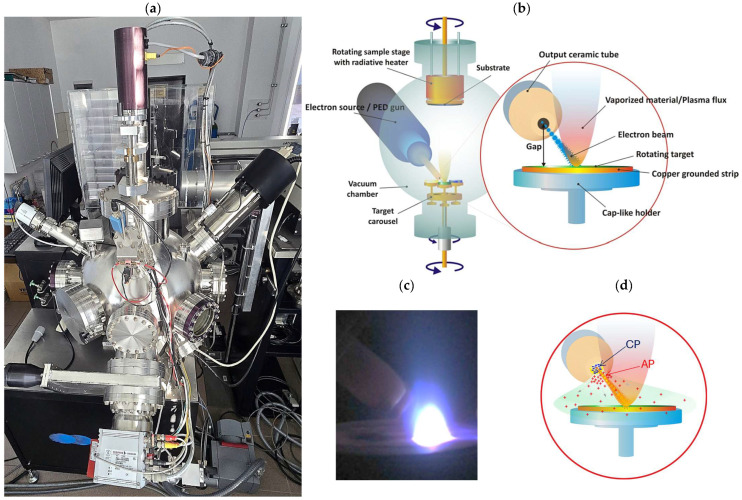
Ablation system built with the PEBS 32 electron gun and the vacuum chamber Pioneer 180 (both Neocera) (**a**). Schematic of the electron deposition process (**b**), a view inside the process chamber (**c**) and a generation of an electron beam (pulse discharge) in the space charge layer formed by the contact between cathode plasma CP and anode plasma AP (**d**).

Pulsed Electron Deposition (PED) technique utilizes a high-energy, pulsed electron beam to vaporize (ablate) material from a target. The ablated material subsequently condenses onto a substrate, forming a coating/film. Due to its unique properties, PED is an attractive alternative to the more widespread method of Pulsed Laser Deposition (PLD). The mechanism of deposition is as follows: a short (approx. 100 ns), intense, high-energy (10–20 keV) electron pulse is generated in an electron source (gun). This pulse strikes the target’s surface, causing its rapid and nearly instantaneous heating and evaporation (Figure 1b,c). In contrast to laser photons, electrons penetrate the material to a finite depth, resulting in volumetric heating. A key requirement of the electron deposition technique is to ensure a uniform and stable distribution of gas pressure (concentration) in the chamber, where the target, as an integral part of the discharge circuit, affects the dynamics of electron beam generation and its parameters (Figure 1d).

Perovskite La(Sr)CoO_3_ thin films were deposited on [001]-oriented, epi-polished single-crystal Si and MgO substrates using pulsed electron deposition. The process was carried out in a Neocera PLD/PEBS 32 system (Neocera, LLC, Beltsville, MD, USA), which is equipped with a Pioneer 180 vacuum chamber and an integrated electron gun (Figure 1a). The key deposition parameters are summarized in Table 1.

The applied parameters successfully produced high-quality, ultra-thin films suitable for subsequent analysis. The electron beam deposition process can be further optimized by slightly reducing the per-pulse electron energy (accelerating voltage) while concurrently increasing the number of pulses to extend the deposition time. Alternatively, a similar effect can be achieved by lowering the pulse repetition rate—a technique frequently recommended for obtaining smooth films on the order of a few nanometers. While increasing the target-to-substrate distance can enhance surface quality, it is important to note that electron ablation is a highly directional process. Therefore, an excessive working distance will negatively impact the film’s homogeneity, particularly its thickness uniformity.

### 3.3. Research and Analytical Techniques Applied

The microstructure and chemical composition of the thin films were analyzed using scanning and transmission electron microscopy. Scanning electron microscopy (SEM) was performed with a FEI Nova NanoSEM 450 system equipped with an EDAX Octane Elect Plus Energy Dispersive Spectroscopy (EDS) detector (Thermo Fisher Scientific Inc., Waltham, MA, USA). Transmission electron microscopy (TEM) analysis was conducted on a JEOL JEM-ARM200F NEOARM microscope (JEOL Ltd., Tokyo, Japan) fitted with an Oxford Instruments EDS detector (Abingdon, Oxfordshire, UK). Cross-sectional samples for TEM were prepared using a focused ion beam (FIB) milling technique.

The crystalline structure of the films was investigated by X-ray diffraction (XRD) on a PANalytical EMPYREAN DY 1061 diffractometer (Malvern Panalytical Inc., Westborough, MA, USA). The analysis was performed in a grazing incidence configuration (α = 1°) using Cu_Kα_ radiation (λ = 0.154 nm). The collected diffraction patterns were interpreted with PANalytical Highscore 4.9 software. Phase identification was carried out using the ICDD PDF-4+ database, while phase analysis and crystallite size determination were performed with the MAUD program (Version 2.9999; 28 May 2025).

The surface topography and roughness of the deposited perovskite thin films were examined by atomic force microscopy (AFM) using a Bruker Dimension Icon instrument (Billerica, MA, USA).

X-ray photoelectron spectroscopy (XPS) was used to analyze surface chemistry. The analyses were performed on a PHI Versa Probe II instrument (Physical Electronics, Inc., Chanhassen, MN, USA) equipped with a monochromatic Al_Kα_ X-ray source (E = 1486.6 eV). Spectra were acquired using a pass energy of 47 eV and a take-off angle of 45°. Charge neutralization was achieved with a combination of an argon ion gun and an electron flood gun. The binding energy scale was calibrated by referencing the C 1s peak of adventitious carbon to 284.8 eV.

**Figure 2:**
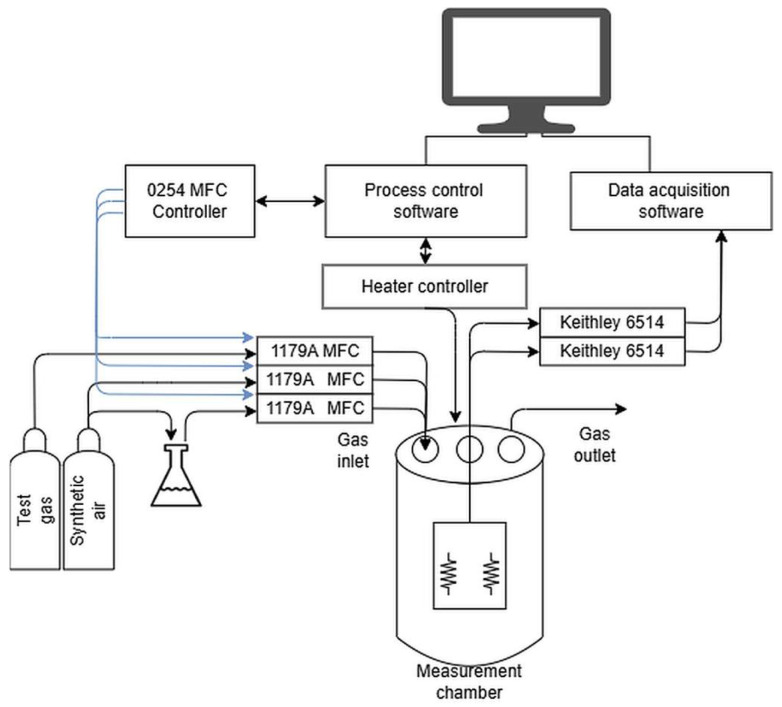
Schematic of the catalytic properties measurement setup; the block diagram illustrates the heated measurement chamber, gas delivery system (including MFCs and bubbler for humidity control), and the resistance measurement setup with Keithley electrometers. The control unit synchronizes temperature regulation, gas flow, and data acquisition.

The catalytic properties measurement system was managed by a computer running dedicated control and data acquisition software GasResControl V1.2 (Figure 2). The electrical resistance of the samples was measured using two Keithley 6514 electrometers (by Keithley Instruments, part of Tektronix, Beaverton, OR, USA), selected for their high accuracy, low noise, and broad measurement range (10 mΩ to 200 GΩ). These instruments provide precise and stable readings critical for evaluating the sensor layers under diverse gas and temperature conditions.

The sensor layers under investigation, deposited on Si substrates, were positioned on a stainless-steel stage within a quartz tube (internal volume: 100 cm^3^) that functions as the measurement chamber. This chamber was equipped with electrical feedthroughs for sensor connections and ports for gas inlet and outlet. Platinum wire contacts were affixed to the sample surface using a conductive silver paste (ELECTON 40AC), which ensures low contact resistance, high thermal stability, and excellent electrical conductivity, particularly at elevated temperatures. The measurement chamber was housed within a furnace where the temperature was precisely maintained by a dedicated PID controller. A temperature probe embedded in the sample stage allowed accurate and direct thermal monitoring.

Gas delivery was managed by four MKS MFC 1179A mass flow controllers (MKS Instruments, Inc., Andover, MA 01810, USA), which provide high precision (±2% of setpoint) and repeatability (±0.2% of full scale) for both test gases and the reference gas (synthetic air). This setup enabled dynamic gas mixing to generate various compositions and humidity levels. Humidity was controlled via a bubbler system, where a stream of synthetic air was passed through water to introduce vapor. The four gas lines—two for test gases, one for synthetic air, and one for humidity control—merged into a single line before the chamber inlet to guarantee a homogeneous gas mixture.

The measurement procedure was computer-controlled and executed in a predefined sequence. Initially, the test chamber was heated to the target setpoint temperature and allowed to stabilize. Subsequently, a gas dosing sequence started, in which test and reference gases were alternately introduced into the chamber. To facilitate rapid gas exchange, a constant total flow rate of 50 sccm was maintained. Throughout the process, electrometers continuously and synchronously recorded the sample’s resistance. The sensor response was defined as the ratio of the layer’s resistance in the test gas to its resistance in the reference gas (synthetic air) at an identical temperature and humidity. To ensure minimal interference in the measurements, high-purity ALPHAGAZ™ 2 gases (Air Liquide, Krakow, Poland) were used, with impurities below 1 ppm for N_2_, O_2_, and Ar, and under 0.1 ppm for hydrocarbons and moisture. The collected resistance data were then processed to determine the sensor’s response characteristics under varying gas concentrations, temperatures, and humidity levels. This method ensures highly controlled and reproducible conditions for evaluating gas-sensing materials, enabling precise characterization of their performance.

## 4. Results and Discussion

### 4.1. XRD Analysis

**Figure 3:**
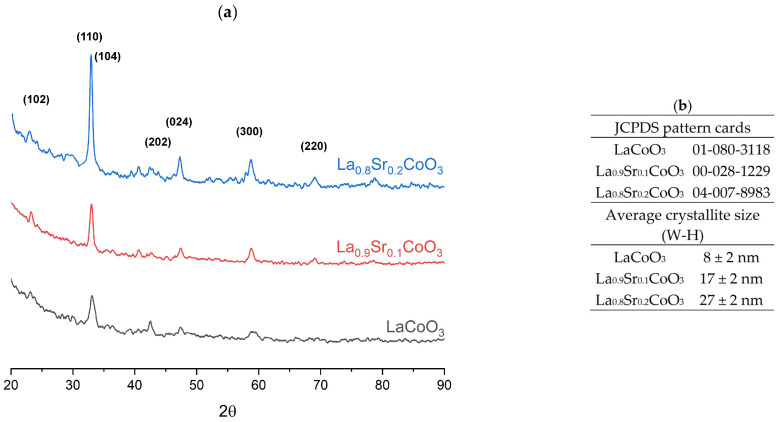
XRD phase analysis of La(Sr)CoO_3_ (**a**) with JCPDS pattern cards and average crystallite sizes (**b**) (estimated using the Williamson-Hall method).

Grazing incidence X-ray diffraction (GIXRD) analysis was performed at an incident angle of α = 1°. Phase identification confirmed the expected structures, corresponding to JCPDS card numbers 01-080-3118 (LaCoO_3_), 00-028-1229 (La_0.9_Sr_0.1_CoO_3_), and 04-007-8983 (La_0.8_Sr_0.2_CoO_3_). As shown in Figure 3, no secondary phases, such as La_2_O_3_, CoO, Co_3_O_4_, or Sr(Co)O_3_, were detected in any of the samples. The high phase purity of the thin films indicates that the pulsed electron deposition process did not induce decomposition in the gas phase, which aligns with theoretical predictions. This suggests that material transfer within the plasma plume occurred in a stoichiometrically congruent manner. These findings highlight the advantages of this ablation technique for depositing chemically and phase-complex materials.

**Figure 4:**
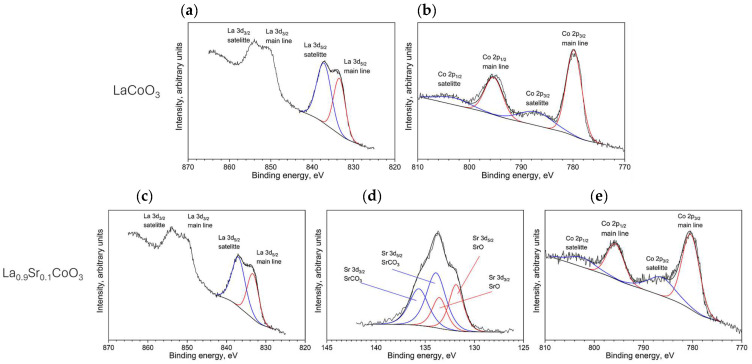
XPS spectra showing the chemical states of elements for La(Sr)CoO_3_ thin films. The top panel displays the La 3d (**a**) and Co 2p spectra (**b**) for LaCoO_3_. The bottom panel displays the La 3d (**c**), Sr 3d (**d**), and Co 2p spectra (**e**) for La_0.9_Sr_0.1_CoO_3_.

The LaCoO_3_ and La(Sr)CoO_3_ films exhibited a preferred (110) orientation, and the intensity of the corresponding diffraction peaks increased with the Sr-dopant concentration (Figure 4a). Compared to the undoped LaCoO_3_, the diffraction peaks for the Sr-doped films shifted to higher 2θ values. This shift resulted from the substitution of La^3+^ ions with Sr^2+^ ions. Although the ionic radii of La^3+^ (1.36 Å) and Sr^2+^ (1.32 Å) are similar, their different valences necessitated charge compensation. This compensation occurred through the oxidation of Co^3+^ to Co^4+^ and/or the formation of oxygen vacancies. The resulting decrease in the cobalt ionic radius—from 0.75 Å for high-spin Co^3+^ to 0.67 Å for high-spin Co^4+^ in octahedral coordination—caused a contraction of the crystal lattice, which explains the observed peak shift.

The X-ray diffraction patterns of the thin films exhibited an elevated background, particularly at low Bragg angles (Figure 3a). This effect is especially prominent for films with thicknesses in the tens of nanometers and stems from two primary sources.

The main contributor is the overwhelming signal from the substrate relative to the film’s very small scattering volume. Because the X-ray beam’s penetration depth of several micrometers far exceeds the film’s thickness, the detected signal is dominated by scattering from the substrate. When an amorphous substrate such as glass is used, it produces a broad amorphous halo that constitutes the primary background noise. This strong halo can effectively mask the weak diffraction peaks originating from the thin film. A secondary factor, inherent to low-angle measurements, is parasitic scatter. This noise arises from the X-ray beam’s interaction with air molecules along its path and with instrument components like collimators and slits, further contributing to the elevated background

The crystallite size was determined using the Williamson-Hall (W-H) method by analyzing the (110), (104), and (024) crystallographic peaks. The average crystallite size for the individual thin films was calculated to be 8 ± 2 nm for LaCoO_3_, 17 ± 2 nm for La_0.9_Sr_0.1_CoO_3_, and 27 ± 2 nm for La_0.8_Sr_0.2_CoO_3_ (as shown in Figure 3b).

### 4.2. XPS Analysis

Figure 4 presents the high-resolution XPS spectra for the La 3d, Co 2p, and Sr 3d core levels of the lanthanum cobaltite thin films. The La 3d spectrum, shown in Figure 4a,c, is characterized by two spin–orbit doublets (3d_5/2_ and 3d_3/2_), despite lanthanum existing in a single oxidation state. The more intense doublet at a lower binding energy (BE ≈ 833 eV) is the main photoemission line, while the second doublet at a higher binding energy is its satellite. This satellite feature is characteristic of lanthanum oxides and perovskites, arising from a final-state effect related to the complex electron configuration (e.g., 3d^9^f^0^L vs. 3d^9^f^1^L, where L denotes a ligand hole). The consistent position and intensity ratio of these doublets across all samples confirm that lanthanum is uniformly in the +3 oxidation state. Similarly, the Co 2p spectrum (Figure 4b,d) consists of a main spin–orbit doublet (Co 2p_3/2_ and Co 2p_1/2_) and a corresponding satellite structure at a higher binding energy. The primary doublet, with the Co 2p_3/2_ peak at BE ≈ 780 eV, is the direct photoemission line, while the satellite feature results from a shake-up process. The position and shape of the Co 2p lines are nearly identical for all films, suggesting that cobalt is in the same chemical state throughout. However, precisely determining its oxidation state is challenging, as the Co 2p binding energy shows minimal shifts between Co^2+^, Co^3+^, and Co^4+^ states. Based on the peak position, the cobalt oxidation state could be +2, +3, or +4. The Sr 3d spectrum, shown in Figure 4e, clearly resolves into two separate spin–orbit doublets, indicating the presence of two distinct strontium species. The doublet at the lower binding energy (BE ≈ 132 eV) is attributed to SrO, while the component at the higher binding energy (BE ≈ 134 eV) corresponds to SrCO_3_. In both compounds, strontium maintains a +2 oxidation state. The presence of strontium carbonate is likely due to a surface reaction between SrO and adventitious carbon, a common surface contaminant [18].

### 4.3. Microstructure of Thin Films (SEM Observation and EDS Analysis)

**Figure 5:**
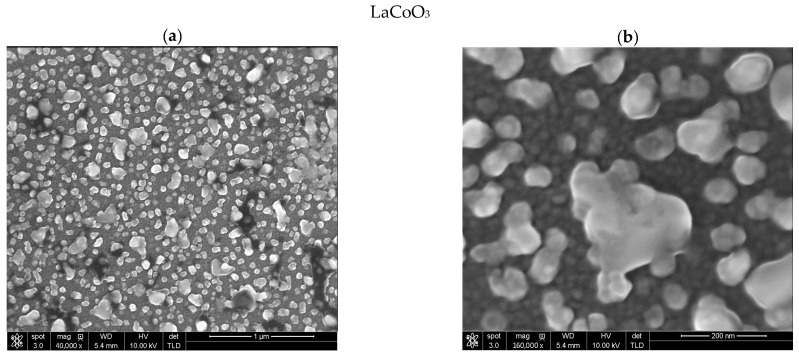

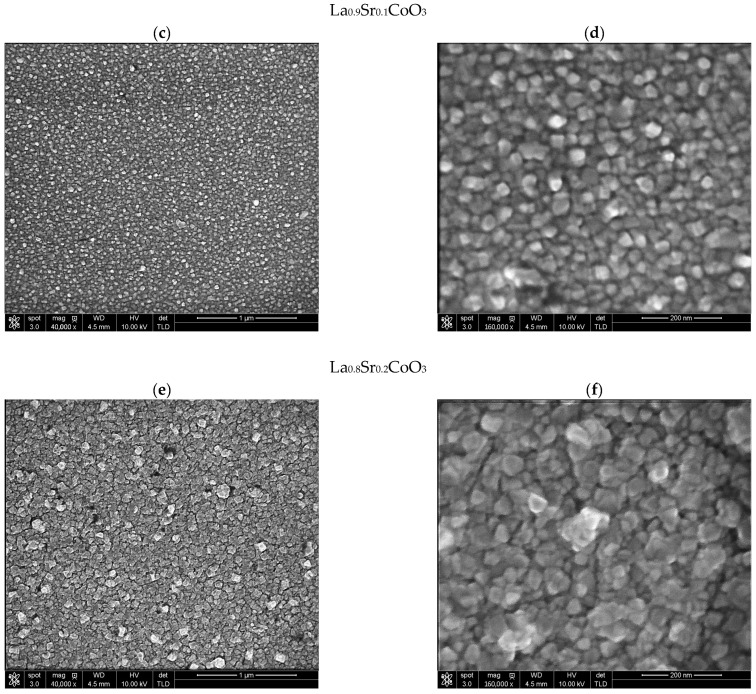
SEM images of the topography of perovskite thin films grown on monocrystalline Si substrates [001] for LaCoO_3_ (**a**,**b**), La_0.9_Sr_0.1_CoO_3_ (**c**,**d**) and La_0.8_Sr_0.2_CoO_3_ (**e**,**f**).

Scanning electron microscopy analysis of the La(Sr)CoO_3_ thin films (Figure 5) reveals that both the stoichiometric perovskite and the Sr-doped films exhibit a compact, dense structure free of surface defects such as pores or cracks. The surfaces of the films occasionally exhibit particle aggregates and droplets; however, their formation is an intrinsic characteristic of the ablation technique. These particulates originate when larger fragments of the target material are ejected and transported to the substrate via the plasma plume, a phenomenon more pronounced at higher energy densities or shorter target-to-substrate distances. Nevertheless, because these features are sparse, they do not substantially degrade the overall quality or functional properties of the film.

Analysis via energy-dispersive X-ray spectroscopy (EDS) verifies that the chemical makeup of these particulates is almost indistinguishable from that of the dense, crystalline film matrix (Table 2). This finding strongly suggests that during electron ablation, decomposition within the gas phase is minimal. As a result, the elemental concentrations in the final film deviate only slightly from the expected stoichiometric ratios.

A primary goal in optimizing the deposition process is to minimize the generation of these particulates. Employing large, smooth targets and carefully tuning the distance between the target and substrate are effective strategies to achieve this. Maintaining an optimal energy density is also critical. If the density is too low, a greater number of pulses is needed to reach the target thickness, risking the formation of unwanted cones on the target surface. Conversely, excessively high energy densities lead to the formation of larger aggregates and a higher incidence of droplets on the film. Consequently, achieving high-quality films hinges on the precise regulation of key process parameters, including laser energy, the partial pressure of oxygen, and the temperature of the substrate. Quantitative EDS analysis was performed using the PhiRhoZ artifact correction method, an approach tailored for thin films. To mitigate the influence of the MgO substrate, the oxygen concentration was determined according to the stoichiometry of the analyzed compound (in Genesis software version 6.53—Type: OxyByDiff).

A key observation for the Sr-doped films is a significant refinement of the surface structure. This indicates that strontium doping not only alters the internal ionic structure but also promotes the formation of smaller crystallites during film growth. These SEM observations are consistent with the crystallite size analysis performed using X-ray diffraction, based on the Williamson-Hall results (Figure 3b), and further suggest that these topographical changes should be observable via atomic force microscopy (AFM).

Stoichiometric LaCoO_3_ (Figure 5a,b) perovskite is characterized by a specific two-layer morphology. A film composed of densely packed, fine crystallites (a few nanometers in diameter) is observed directly on the silicon substrate, upon which significantly larger crystalline aggregates subsequently grow. This structure strongly indicates a change in the thin film’s growth mechanism after it reaches a critical thickness, which is related to a shift in thermodynamic conditions at the surface. This suggests a transition from a layer-by-layer growth, described by the Frank-van der Merwe (FM) model [19], to a layer-plus-island mechanism, known as the Stranski-Krastanov (SK) model [20].

Frank-van der Merwe (FM) model describes an ideal, two-dimensional (2D) “layer-by-layer” growth. In this mechanism, the atoms of the deposited layer bond more strongly to the substrate atoms than to each other, leading to the formation of smooth, continuous films. Each new atomic layer begins to crystallize only after the preceding one is fully complete. The conditions for such growth are strong adhesion between the deposited material and the substrate, and a minimal mismatch between their crystal lattices. This is the desired growth model for producing high-quality epitaxial layers, for instance, in semiconductor technology [21].

The Stranski-Krastanov (SK) model is an intermediate mechanism. Initially, growth proceeds according to the FM model, forming one or more complete atomic layers known as the wetting layer. However, after reaching a critical thickness, the strain accumulating in the film—resulting from the crystal lattice mismatch—triggers a shift to a three-dimensional (3D) island-like growth. This model plays a key role in the self-assembly of nanostructures, such as quantum dots [22]. In the case of La(Sr)CoO_3_ perovskite, this mechanism leads to significant topographical development of the surface. This is a desirable phenomenon, as it enhances the material’s catalytic activity by generating a greater number of chemically active sites [23].

This transition between growth mechanisms can be explained by a change in surface energy that occurs after the formation of the wetting layer during the initial stage of the deposition process by spark discharge (electron ablation).

### 4.4. Topographic Analysis of Thin Film Surfaces (AFM Study and Roughness Parameter Measurements)

**Figure 6:**
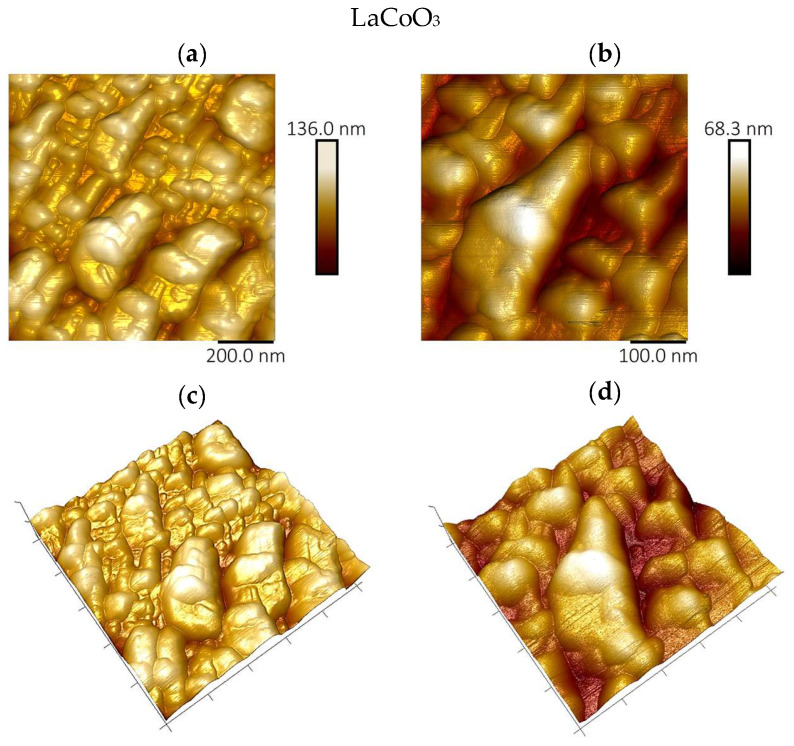
Surface topography images of perovskite thin films by atomic force microscopy technique for LaCoO_3_; imaging area 1 μm^2^ (**a**,**c**) and 0.25 μm^2^ (**b**,**d**).

**Figure 7:**
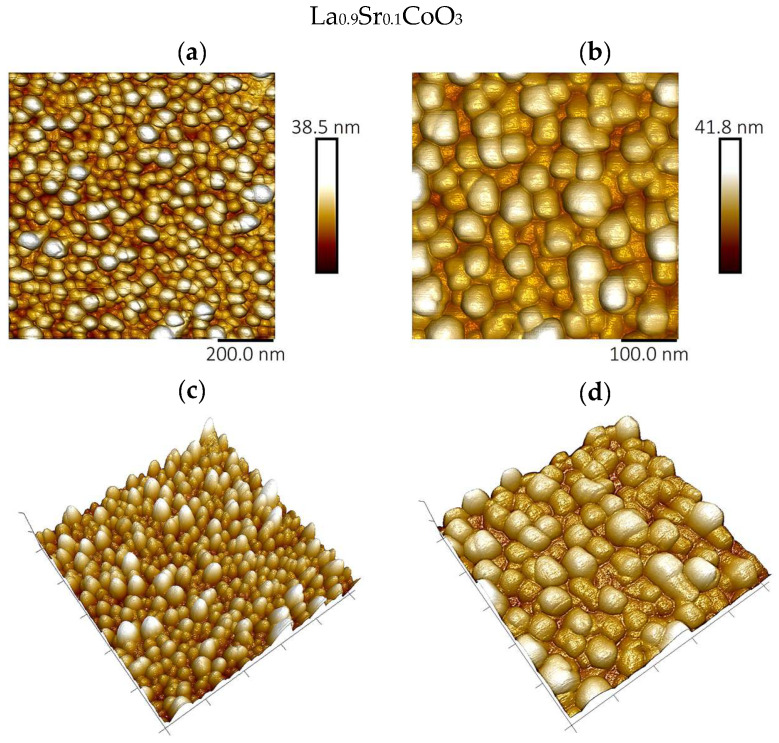
Surface topography images of perovskite thin films by atomic force microscopy technique for La_0.9_Sr_0.1_CoO_3_; imaging area 1 μm^2^ (**a**,**c**) and 0.25 μm^2^ (**b**,**d**).

**Figure 8:**
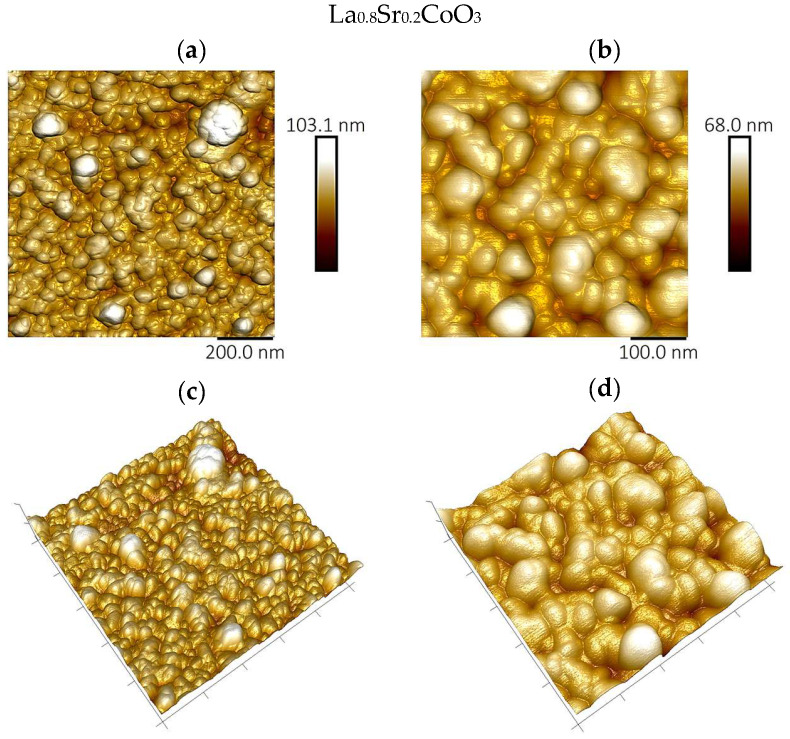
Surface topography images of perovskite thin films by atomic force microscopy technique for La_0.8_Sr_0.2_CoO_3_; imaging area 1 μm^2^ (**a**,**c**) and 0.25 μm^2^ (**b**,**d**).

Atomic force microscopy analysis of the La(Sr)CoO_3_ thin films, performed in air using tapping mode, corroborates the larger-scale observations from scanning electron microscopy, as shown in Figure 6, Figure 7 and Figure 8. The film surfaces consist of fine, irregular grains. In certain areas, particularly on the La_0.8_Sr_0.2_CoO_3_ sample, larger conglomerates with diameters of less than 1 μm are present.

The AFM images of the Sr-doped LaCoO_3_ thin films (Figure 7 and Figure 8) confirm previous SEM findings, clearly revealing that the surface structure becomes significantly fragmented as Sr doping progresses. Moreover, the doped La(Sr)CoO_3_ perovskites display a markedly homogenized surface topography, with features that are more regular and rounded than the irregular surface morphology characteristic of stoichiometric LaCoO_3_ (Figure 6). These qualitative observations are quantitatively substantiated by the roughness parameters compiled for all perovskites in Table 3.

A clear trend emerges when comparing the films’ roughness. For La(Sr)CoO_3_ thin films, the changes in surface morphology and topography are reflected in a reduction in the roughness parameters (Table 3). In contrast, for the undoped LaCoO_3_, the apparent surface changes associated with the previously described switch in growth mechanism—from an ultrafine, uniform wetting layer to island-like conglomerates—result in a slight increase in roughness. Despite these variations, the overall roughness values indicate that all perovskite films are smooth, as the topographical changes are on the order of a few nanometers. Such a topography is typical for ablation techniques (electron or laser) and is consistent with reports on other functional oxides prepared by PLD, including (Co,Ca)O [24] and La(Sr)FeO_3_ thin films [25,26].

### 4.5. TEM Microstructure Investigation

**Figure 9:**
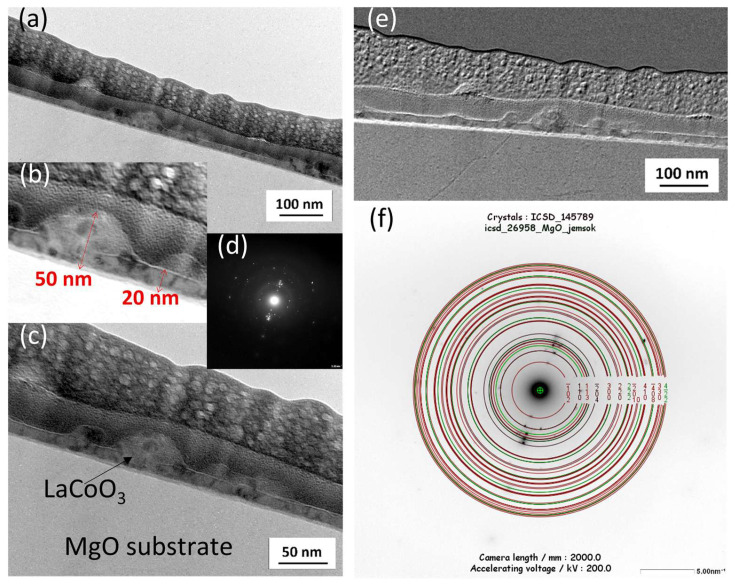
Cross-sectional transmission electron microscopy analysis of a LaCoO_3_ thin film: (**a**,**c**) low-magnification bright-field (BF) TEM images, (**b**) magnified view of the selected area from (**a**–**d**) selected area electron diffraction pattern, with (**f**) its corresponding solution, (**e**) STEM-SAAF image of the same area.

**Figure 10:**
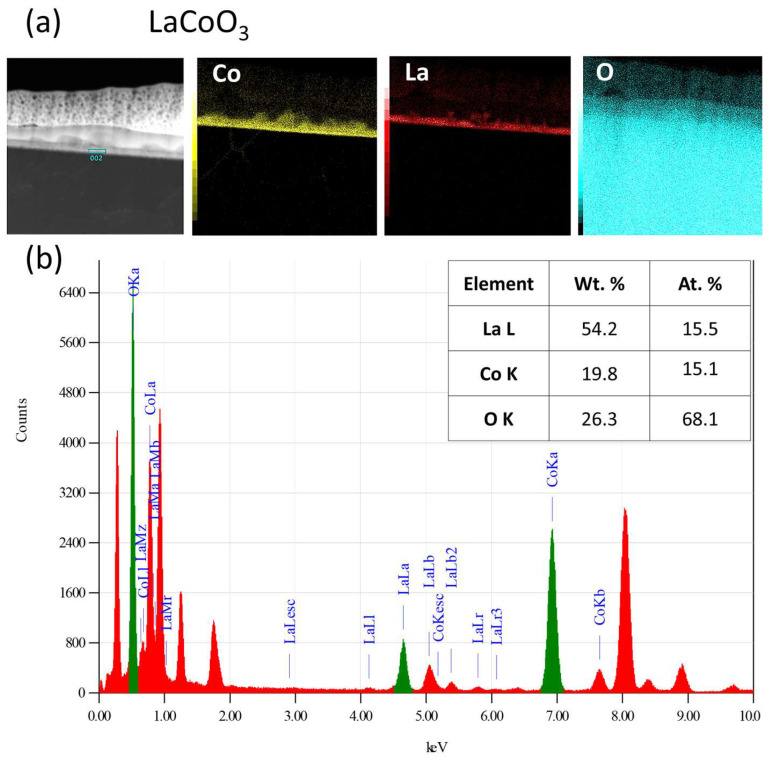
TEM/EDS analysis of LaCoO_3_ thin film: (**a**) distribution maps of key elements. (**b**) EDS spectrogram showing peaks from the characteristic spectral lines of the analyzed elements, along with the averaged results of the quantitative analysis.

**Figure 11:**
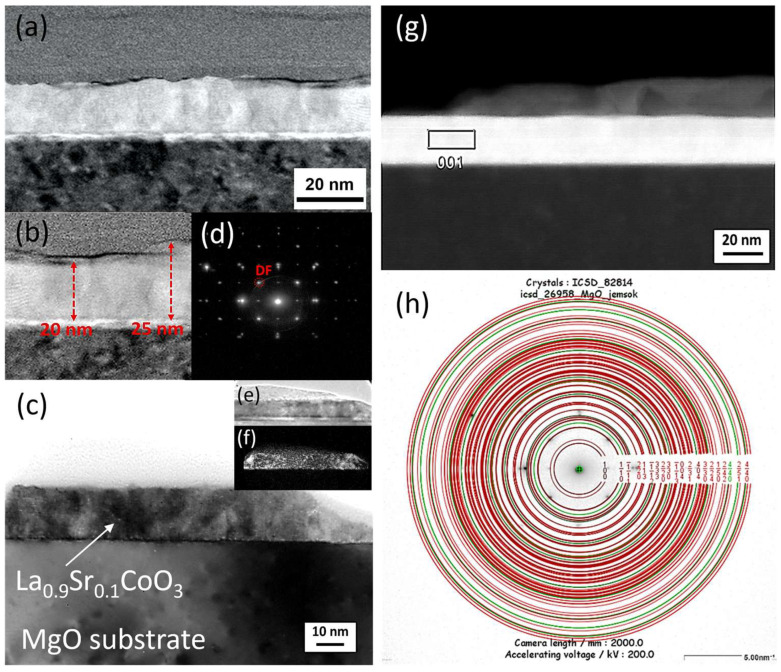
Cross-sectional transmission electron microscopy analysis of a La_0.9_Sr_0.1_CoO_3_ thin film: (**a**,**c**,**g**) low-magnification bright-field (BF) TEM images, (**b**) magnified STEM-SAAF image of the same area, (**d**) selected area electron diffraction pattern, with (**e**,**f**) bright and dark-field TEM images and (**h**) its corresponding solution.

**Figure 12:**
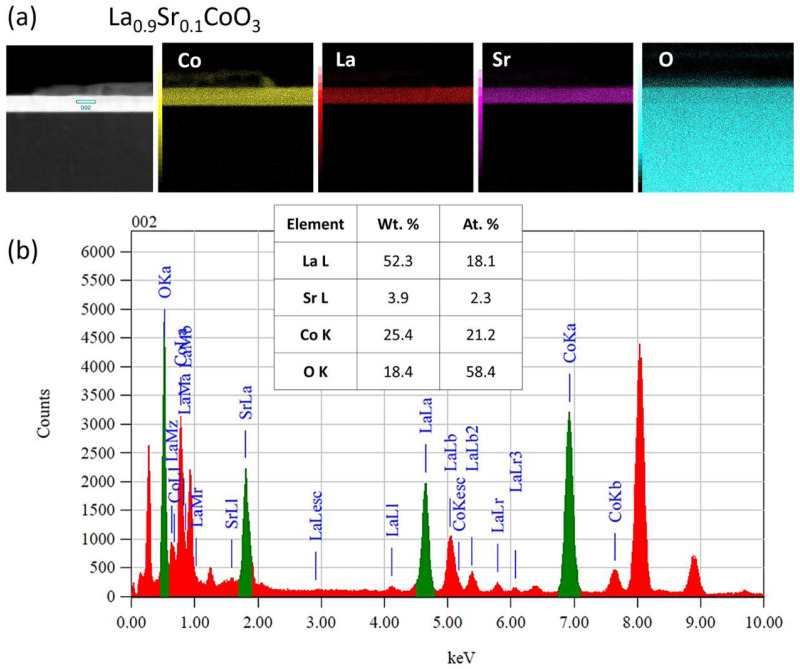
M/EDS analysis of La_0.9_Sr_0.1_CoO_3_ thin film: (**a**) distribution maps of key elements. (**b**) EDS spectrogram showing peaks from the characteristic spectral lines of the analyzed elements, along with the averaged results of the quantitative analysis.

**Figure 13:**
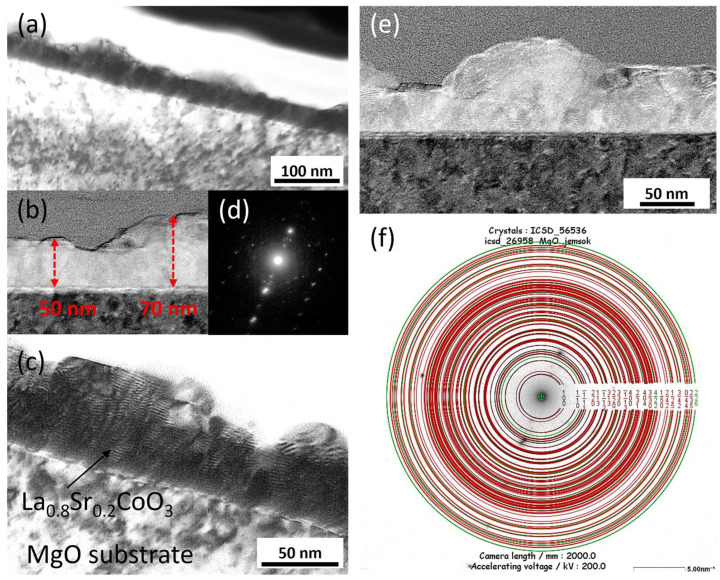
Cross-sectional transmission electron microscopy analysis of a La_0.8_Sr_0.2_CoO_3_ thin film: (**a**,**c**) low-magnification bright-field (BF) TEM images, (**b**) magnified view of the selected area from (**a**,**d**) selected area electron diffraction pattern, with (**f**) its corresponding solution, (**e**) STEM-SAAF image of the same area.

**Figure 14:**
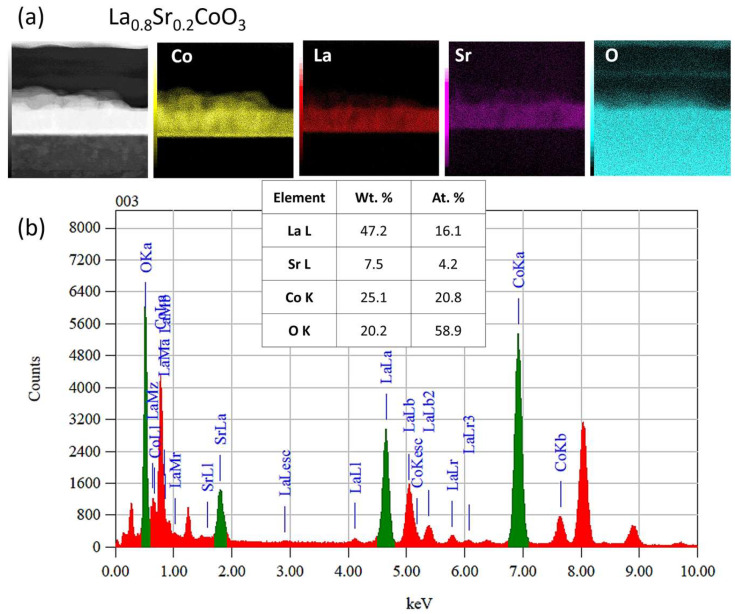
TEM/EDS analysis of La_0.8_Sr_0.2_CoO_3_ thin film: (**a**) distribution maps of key elements. (**b**) EDS spectrogram showing peaks from the characteristic spectral lines of the analyzed elements, along with the averaged results of the quantitative analysis.

Cross-sectional transmission electron microscopy analysis, which combined conventional TEM/STEM bright/dark-field imaging with selected area electron diffraction (SAED), revealed that all LaCoO_3_ and La(Sr)CoO_3_ thin films possessed an ultra-fine nanocrystalline structure. This structure featured local irregularities (i.e., conglomerates), which is characteristic of the Pulsed Electron Deposition method and consistent with prior SEM/AFM surface observations (Figure 9, Figure 10, Figure 11, Figure 12, Figure 13 and Figure 14. The stoichiometric LaCoO_3_ (Figure 9 and Figure 10), Sr-doped La_0.9_Sr_0.1_CoO_3_ (Figure 11 and Figure 12)) and La_0.8_Sr_0.2_CoO_3_ (Figure 13 and Figure 14) films exhibited dense, columnar crystallites with well-defined boundaries that grew directly from the Si/MgO substrate interface. This internal structure is a consequence of the layer-by-layer (Frank-van der Merwe, FM) growth mechanism inherent to the PED process. Growth begins with the formation of an initial wetting layer—approximately 20 nm thick for LaCoO_3_ and La_0.9_Sr_0.1_CoO_3_, and around 50 nm for La_0.8_Sr_0.2_CoO_3_. Within this region, the films are extremely compact, a morphology dictated by the stronger adhesion of atoms/ions to the substrate than to each other. The total thickness of the perovskite films ranged from 20 to 70 nm (Figure 9b, Figure 11b and Figure 13b). Based on these values, the average size of the surface conglomerates (“islands”) was estimated to be 20–30 nm. Consequently, the deposition rate for all stoichiometric and Sr-doped LaCoO_3_ thin films was calculated to be approximately 1–3.5 × 10^−4^ nm/pulse.

Selected area electron diffraction patterns confirmed the presence of only the desired crystalline phases, consistent with previous XRD analyses: LaCoO_3_ (Figure 9d,f), La_0.9_Sr_0.1_CoO_3_ (Figure 11d,h), and La_0.8_Sr_0.2_CoO_3_ (Figure 13d,f). However, analyzing such thin structures (20–70 nm) is often prone to error from signal interference from the substrate, potential intermediate layers, and elements (Pt, C) intentionally introduced during FIB sample preparation. For this reason, reflections from the MgO substrate (green) were deliberately left in the indexed diffraction patterns alongside those from the perovskite (red) for reference (Figure 9f, Figure 11h and Figure 13f).

Furthermore, all examined La(Sr)CoO_3_ perovskite films were found to be chemically stable and homogenous. This was clearly indicated by both qualitative analysis (elemental distribution maps for La, Sr, Co, and O) and quantitative analysis performed using the TEM-EDS technique (Figure 10, Figure 12 and Figure 14).

### 4.6. Electrical Resistance Measurements

**Figure 15:**
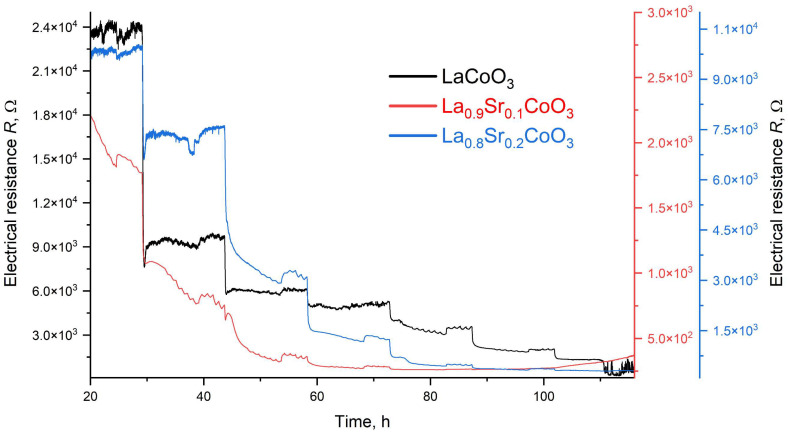
Response of La(Sr)CoO_3_ exposed to 10, 50 and ppm NO_2_ at temperatures in the range 100–450 °C.

The gas sensing properties of thin films of LaCoO_3_ perovskite and its Sr-doped variants were evaluated for NO_2_ detection at concentrations of 10, 50, and 100 ppm over an operating temperature range of 100–450 °C (Figure 15).

Prior to measurements, each sensor was pretreated in the test chamber by heating to 300 °C for at least three hours under a continuous flow of synthetic air to remove surface and chamber contaminants. Subsequently, for each measurement cycle, the sensor was set to the target operating temperature and held under a constant applied voltage and continuous air flow until a stable baseline resistance (R_air_) was achieved. The sensing performance was then evaluated by cycling the gas environment between clean air and the target NO_2_ concentration, maintaining a constant total flow rate of 200 cm^3^ min^−1^ and a relative humidity of 50% ± 2%.

**Figure 16:**
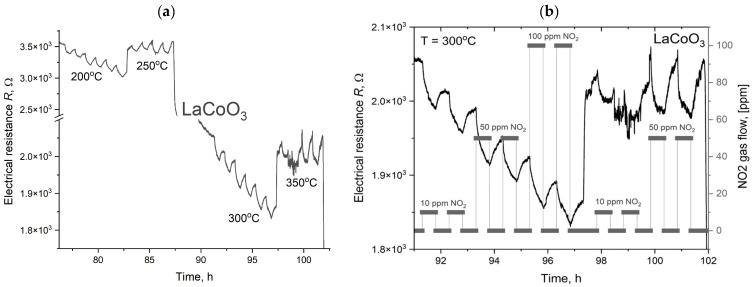
Response of LaCoO_3_ exposed to 10, 50 and ppm NO_2_ at temperatures in the range 200–350 °C (**a**) with an enlarged area for the 300 °C (**b**).

**Figure 17:**
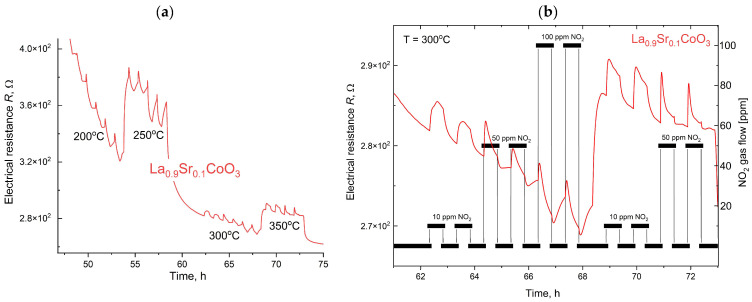
Response of La_0.9_Sr_0.1_CoO_3_ exposed to 10, 50 and ppm NO_2_ at temperatures in the range 200–350 °C (**a**) with an enlarged area for the 300 °C (**b**).

**Figure 18:**
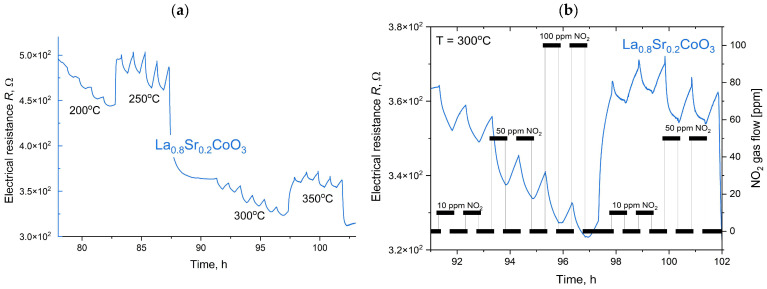
Response of La_0.8_Sr_0.2_CoO_3_ exposed to 10, 50 and ppm NO_2_ at temperatures in the range 200–350 °C (**a**) with an enlarged area for the 300 °C (**b**).

As shown in Figure 15, Figure 16, Figure 17 and Figure 18, the baseline resistance of the films decreased as the operating temperature increased, which is characteristic behavior for a p-type semiconductor material. This trend is attributed to the thermal generation of charge carriers, which increases the material’s conductivity. Upon exposure to NO_2_, a distinct decrease in resistance was observed at all tested temperatures. This response is consistent with the behavior of a p-type perovskite. The adsorption of oxidizing NO_2_ molecules on the sensor surface increases the concentration of majority charge carriers (holes), thereby decreasing the overall resistance of the film. The graph summarizing data for various compositions of La(Sr)CoO_3_ perovskite thin films across the entire temperature range (Figure 15) shows that strontium Sr-doping enhances catalytic stability, especially at elevated temperatures. The three curves were scaled appropriately to allow for a direct comparison.

The sensing response (Resp) and sensitivity (S) of the thin films to NO_2_ were calculated from the experimental data using the following equations:

Resp = R_NO_2__/R_air_, (1)

S = ((R_NO_2__ − R_air_)/R_air_)·100%, (2)

where R_NO2_—stable measured electrical resistance in NO_2_ atmosphere; R_air_—stable measured electrical resistance in air.

**Figure 19:**
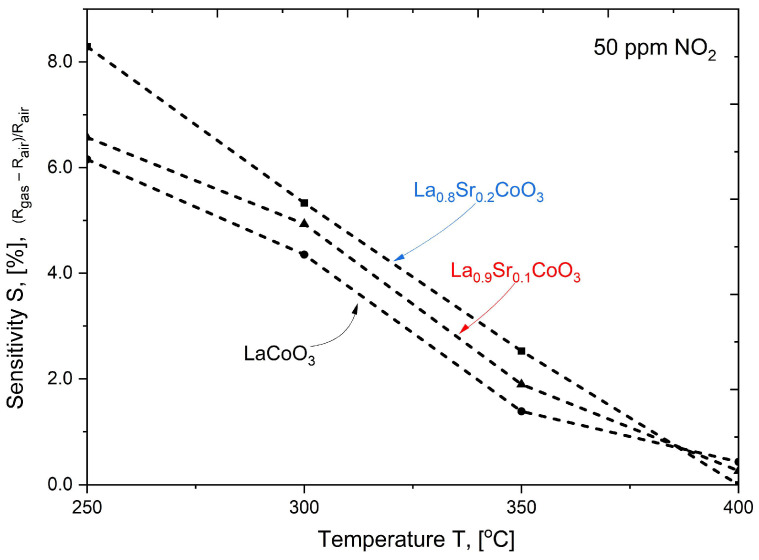
Sensitivity changes in La(Sr)CoO_3_ thin films to 50 ppm NO_2_ in the 250–400 °C temperature range.

A summary of the calculation results for the La(Sr)CoO_3_ thin films is provided in Table 4, with a graphical representation presented in Figure 19. The analysis of electrical resistance changes over time for thin La_1−x_Sr_x_CoO_3_ films at various temperatures (see Figure 19 and Table 4) yields several key findings. Across all strontium doping levels, the response (Resp) to both NO_2_ and air is stable, ranging from 1.0 to 1.1. However, a significant drawback is the consistently long response and recovery times (t_res_, t_rec_), which are approximately 29–30 min. A comparison of the films reveals that while the undoped LaCoO_3_ and the La_0.9_Sr_0.1_CoO_3_ films (Figure 16 and Figure 17) exhibit similar sensitivity (S) changes, the La_0.8_Sr_0.2_CoO_3_ film (Figure 18) demonstrates the most promising characteristics. Despite its long response and recovery times, this film shows enhanced operational stability in the 200–400 °C temperature range and an increasing sensitivity (by approximately 20%) at temperatures up to 350 °C. These results strongly suggest that a strontium doping level (around 7 Wt.%) significantly enhances the catalytic properties of lanthanum cobaltite La(Sr)CoO_3_ thin films synthesized via Pulsed Electron Deposition. This conclusion aligns well with previous findings reported for perovskite films of the same composition prepared by Pulsed Laser Deposition [26].

The kinetics of Sr-doped LaCoO_3_ perovskite thin films, specifically their response and recovery times, are governed by an intricate relationship involving surface structure, gas adsorption–desorption processes, and reaction rates. Strontium doping fundamentally modifies these characteristics by inducing the formation of oxygen vacancies, with concentrations proportional to the Sr content, and by altering the oxidation states of the cations. After accounting for the structural properties of the synthesized films and postulating a uniform interaction mechanism at the active surface sites, the disparities in performance can be ascribed to differences in surface morphology. Indeed, the increased surface granulation of La(Sr)CoO_3_, corroborated by SEM/AFM imaging and roughness data, is associated with faster response and recovery, particularly in the La_0.8_Sr_0.2_CoO_3_ composition. A reduction in crystallite size, combined with a typical island-like growth mechanism, refines the surface topography of the thin film. This refinement boosts the concentration of chemically active centers, which is key to enhancing the film’s sensitivity and stability. Consequently, these findings show that optimizing the structure of the gas-sensitive material is crucial for achieving high catalytic performance.

## 5. Summary and Discussion

La(Sr)CoO_3_ perovskites exhibit enhanced sensitivity to a broad range of gases, including volatile organic compounds (VOCs), nitrogen oxides, and carbon monoxide. Their unique crystal structure and surface properties facilitate strong interactions with gas molecules, inducing significant changes in electrical conductivity. In contrast, conventional metal oxides—typical sensor materials—often show limited sensitivity or require high operating temperatures, which restricts their practical application.

A key advantage of perovskites is that their selectivity toward specific gases can be finely tuned through compositional modifications, such as doping. This tunability stems from the ability to alter the oxygen binding energy and engineer oxygen vacancies within the crystal lattice, creating active sites for selective interactions. Achieving a comparable level of selectivity with traditional metal oxides is considerably more challenging due to their limited compositional and structural flexibility.

Perovskite-based sensors offer distinct advantages over traditional metal oxide sensors, particularly in terms of compositional flexibility, operating temperature, and response kinetics. The versatile perovskite crystal structure allows for a high degree of compositional flexibility by accommodating a wide range of metallic and non-metallic elements within its lattice. This adaptability enables the precise tuning of the sensor’s properties to meet the requirements of specific applications. In contrast, traditional metal oxides typically possess a more rigid stoichiometry, which inherently limits the flexibility of sensor design. Perovskite-based sensors can operate stably at significantly lower temperatures compared to their traditional oxide counterparts. This characteristic drastically reduces power consumption and extends the sensor’s operational lifespan. Conversely, traditional metal oxides often necessitate high operating temperatures to achieve optimal performance, which leads to greater energy demands and can introduce long-term stability concerns. Perovskites frequently demonstrate faster response and recovery times to fluctuations in gas concentration. This rapid kinetic response is critical for applications requiring real-time monitoring of dynamic environments. The slower kinetics of traditional oxide sensors, however, can limit their effectiveness and utility in such scenarios.

Modeling the electrical resistance of La(Sr)CoO_3_ perovskite thin films with varying strontium concentrations presents a complex problem. This complexity is primarily driven by changes in the material’s ionic structure, especially the formation of oxygen vacancies. Such modifications can compromise the integrity of the fundamental perovskite structure, a significant effect observed even with low Sr dopant levels of only a few atomic percent. Therefore, this study deliberately utilized Sr concentrations engineered to achieve the specific stoichiometries of La_0.9_Sr_0.1_CoO_3_ and La_0.8_Sr_0.2_CoO_3_. The successful synthesis of these compositions was confirmed through the material analyses (XRD, SEM/EDS, TEM, AFM, and XPS) presented in earlier chapters.

The concentration of the strontium (Sr) dopant is critical to the material’s properties; however, both excessively low and high levels present challenges. At low concentrations (1–5% Sr), obtaining significant and repeatable changes in resistance is difficult, as the substrate can interfere with the measurements. Conversely, excessive doping (approaching or exceeding 40–50% Sr) causes significant distortions in the cation sublattice and increases the risk of surface defects [27]. These defects can generate unfavorable tensile stresses that counteract the benefits of increasing the number of chemically active surface sites, which is crucial for catalytic applications. As a result, structural changes dominate the measurements, leading to significant errors. Based on the stoichiometry of the obtained films, Sr dopant concentrations in the 4–10% range were selected for this study. This range demonstrated significant changes in conductivity (resistivity in the presence of NO_2_), which will allow for a planned, systematic narrowing of this window in future research. The increased strontium concentration modifies the resistance by affecting the hopping of charge carriers between cobalt (Co) ions of different valences, as discussed in the XRD analysis. It is also worth noting that at higher temperatures (above 500 °C), polaron transport may become the dominant mechanism, particularly in materials with a high defect concentration (reaching several tens of percent). These observations suggest an optimal strontium doping range of a few percent to approximately 15%. This range provides a balance between high conductivity and the preservation of a compact structure, homogeneity, and controlled surface roughness. Exceeding or falling below this range can lead to uncontrolled growth in the material’s resistance.

The electrical properties of La(Sr)CoO_3_, particularly its resistance, are highly dependent on external factors, most notably temperature. This relationship, which is incorporated into our modeling, was investigated over a broad measurement range of 100–450 °C. This range corresponds to the observed, repeatable changes in the material’s catalytic properties in response to specific external stimuli. Acknowledging this is crucial for potential applications in resistive sensors. In such devices, size and weight constraints necessitate the use of lightweight, chemically resistant substrate materials (e.g., light non-ferrous metal alloys or polymer-based composites), which practically limits the operating temperature to below 500 °C.

A key challenge for the long-term application of Sr-doped LaCoO_3_ thin films as gas sensors is their degradation and the alteration of their catalytic properties during operation. The degradation process is complex and depends on multiple factors, including environmental conditions (e.g., temperature, atmosphere) and thermal cycling. High temperatures, often exceeding the material’s recommended operating range, accelerate degradation mechanisms such as ion diffusion, phase changes, and oxidation. The presence of moisture, CO_2_, SO_x_, and other corrosive gases can induce reactions with the perovskite, leading to adverse changes in its composition and structure. Furthermore, repeated temperature fluctuations can generate mechanical stress, causing cracking and delamination of the film. Consequently, this study does not focus on the predictive modeling of structural changes but rather on verifying the actual layer growth conditions to obtain ideal structures. The goal is to produce films with properties that correlate with the predicted changes in catalytic activity in the presence of NO_2_ within a specific temperature range. A thorough understanding of these factors is essential for optimizing the electrical properties of La(Sr)CoO_3_ thin films for their use in electronic and electrochemical devices.

To enhance the response and reaction times of thin-film La(Sr)CoO_3_ perovskite sensors, optimizing their topography and minimizing ionic structural defects are critical. Both characteristics directly influence analyte diffusion rates, charge transport, and overall sensor performance. The topography of the sensing film—including its porosity, grain size, and total surface area—plays a fundamental role in determining sensor reaction time. A more porous or nanostructured surface, such as those with nanotube or nanorod architectures, offers a higher surface-area-to-volume ratio. This facilitates faster and more efficient adsorption of analyte molecules, directly shortening the response time. However, a high density of grain boundaries in polycrystalline films can act as trapping centers for charge carriers, thereby slowing the sensor’s reaction. This appears to be the primary cause of the extended response and reaction times observed in our research (~30 min), despite the films’ stability. Fabricating films with slightly larger, well-interconnected nanocrystallites can streamline charge transport and accelerate response times. Film thickness is also a key parameter. An excessively thick film can impede the diffusion of the analyte to active sites, whereas a film that is too thin may not offer enough interaction sites due to its limited topographical development.

Defects within the perovskite crystal lattice, such as ion vacancies, critically affect the sensor’s electronic properties and reaction time. These imperfections promote the formation of localized energy states within the band gap, which can trap electrons and holes. This trapping mechanism slows their transport to the electrodes and lengthens the time required to generate a measurable signal. Furthermore, these defects often serve as non-radiative recombination centers, reducing the number of charge carriers that contribute to the sensory signal. This can decelerate both the response and recovery times. Additionally, the mobility of ionic defects under an electric field can lead to signal instability and hysteresis, complicating accurate and rapid measurements. Based on these findings, we propose two primary modification strategies for the La(Sr)CoO_3_ thin films analyzed in this study. Compositional engineering—refining the doping range to precisely tune the material’s stoichiometry. This should be based on the two most promising doping variants identified in our work (La_0.9_Sr_0.1_CoO_3_ and La_0.8_Sr_0.2_CoO_3_), focusing on films that have already demonstrated superior structural characteristics. Precise control over the substrate’s annealing temperature and duration, both during the pre-deposition cleaning and the post-deposition stages, is crucial. This will allow for targeted management of grain growth and the final microstructure of the film.

The degradation of thin-film perovskite films in gas sensors exposed to nitrogen dioxide (NO_2_) is a complex chemical and structural process. The primary cause is the highly oxidizing nature of the NO_2_ molecule, which reacts with the perovskite surface, leading to irreversible damage and a loss of sensing capabilities. The degradation mechanism is a multi-stage process driven by several key phenomena. As strong electron acceptors (oxidants), NO_2_ molecules first adsorb onto the perovskite surface and withdraw electrons from its crystal lattice. This removal of electrons destabilizes the delicate ionic balance within the perovskite structure, often leading to the material’s decomposition. This process forms new surface phases that are inactive for sensing, causing permanent changes to the layer’s morphology and electronic properties. Consequently, the sensor exhibits reduced sensitivity, slower response and recovery times, and poor long-term stability. The degradation process is significantly accelerated by the presence of moisture. Water (H_2_O) can facilitate chemical reactions on the perovskite surface and promote the formation of hydrated intermediate forms. These hydrated perovskites are typically unstable and decompose readily, hastening the overall decay of the sensor material.

Several strategies are employed to increase the stability of perovskite gas sensors and their resistance to NO_2_. One primary approach is chemical composition engineering. Modifying the material’s formula—for example, by replacing an unstable cation or creating mixed, non-stoichiometric compositions—can significantly improve the thermal and chemical stability of the perovskite structure. Another effective method is the synthesis of two-dimensional (2D) or mixed quasi-2D/3D structures. In these architectures, long organic molecules act as natural barriers, shielding the perovskite material from moisture and aggressive gases and thereby extending its operational lifetime. In this context, the applied electron ablation method appears to be highly encouraging, as shown by the present structural studies. Finally, morphology control is crucial. The creation of dense, uniform perovskite layers with large, well-defined crystallites minimizes the number of grain boundaries. Since grain boundaries are preferential sites for initiating degradation, a high-quality film morphology enhances the material’s overall robustness. By combining these strategies, it is possible to significantly extend the service life and improve the reliability of thin-film perovskite NO_2_ sensors, moving them closer to practical commercial applications.

Strontium-doped LaCoO_3_ perovskite thin films show significant potential for various applications, particularly in gas sensing (for NO_x_, CO, and VOCs), environmental monitoring, and as temperature and humidity sensors. Sr doping enhances the material’s conductivity and reactivity, leading to improved sensitivity and selectivity. The key mechanism involves the introduction of Sr dopant into the LaCoO_3_ structure, which allows for the control of oxygen ion mobility by generating oxygen vacancies responsible for the material’s chemical activity. Despite these advantages, thin films are particularly susceptible to degradation due to their large surface area in contact with the environment. Typical degradation processes include ion diffusion, reactions with ambient gases (e.g., moisture and CO_2_, leading to the formation of carbonates or hydroxides), chemical poisoning of active sites, and high-temperature grain sintering, which reduces the active surface area. These phenomena manifest as a significant decrease in catalytic activity, a deterioration in selectivity and stability, and a shortened film lifespan. To counteract degradation, it is crucial to fabricate films with a high-quality internal structure—specifically, a dense, columnar, and defect-free crystal morphology. Such conditions can be achieved through controlled epitaxial growth using Pulsed Electron Deposition. The material analyses presented in this work confirm the effectiveness of an optimized PED process for producing high-quality La(Sr)CoO_3_ thin films. This achievement is particularly noteworthy because electron beam ablation is often considered more challenging to control and less reproducible than the more common pulsed laser deposition technique. Therefore, optimizing the perovskite’s chemical composition and structure through a well-defined fabrication process is essential for limiting degradation and ensuring long-term stability.

Thin films of LaCoO_3_ perovskite are fabricated using various methods, including magnetron sputtering, atomic layer deposition (ALD), molecular beam epitaxy (MBE), and physical vapor deposition (PVD) based ablative techniques such as pulsed laser deposition (PLD) and pulsed electron deposition (PED). Ablative techniques are particularly advantageous as they minimize gas-phase decomposition, ensuring a precise stoichiometric transfer from the target to the substrate when optimized. Furthermore, these methods offer exceptional control over film thickness by adjusting parameters like power density, working distance, and the number of pulses. This precision is critical for the seamless integration of La(Sr)CoO_3_ films with microelectromechanical systems (MEMS), facilitating miniaturization and integration with other electronic components. From a manufacturing perspective, ablative techniques are scalable and relatively cost-effective. This financial advantage is amplified by the low cost of perovskite materials, making the approach well-suited for the mass production of thin films. Moreover, recent developments in fabricating these films on flexible substrates are paving the way for novel applications, such as wearable sensors integrated into everyday textiles.

## 6. Conclusions

This study investigates the structural, morphological, and electrical properties of pure and Sr-doped lanthanum cobaltite perovskite thin films. Fabricated using pulsed electron deposition, these films were evaluated for their potential application as active electrodes in nitrogen dioxide (NO_2_) gas sensors operating in a range of 200 °C to 450 °C. The key findings are summarized as follows:

1. High-Quality Film Deposition: The electron deposition technique successfully produced high-quality, nanocrystalline La(Sr)CoO_3_ thin films on Si and MgO substrates. The films exhibited a compact, columnar structure with a low defect density, confirming the efficacy of electron ablation for this purpose. Cross-sectional analysis revealed a transition in the growth mechanism from a two-dimensional, layer-by-layer model to a three-dimensional island growth, which refined the surface topography.
2. Influence of Sr Doping: The introduction of Sr as a dopant significantly altered the microstructure and surface topography of the films. As confirmed by XRD, SEM, and AFM analyses, doping promoted the formation of smaller crystallites and a more uniform surface, an effect particularly evident in the La_0.8_Sr_0.2_CoO_3_ film.
3. Gas Sensing Performance: The La(Sr)CoO_3_ films demonstrated a distinct electrical response to NO_2_ gas. The La_0.8_Sr_0.2_CoO_3_ composition, in particular, showed highly promising sensing properties, characterized by excellent stability and enhanced sensitivity to NO_2_ at operating temperatures up to 350 °C. Furthermore, these doped films exhibited slightly faster response and recovery times compared to their undoped counterparts.

In conclusion, this research validates the potential of Sr-doped LaCoO_3_ thin films as effective sensing electrodes for NO_2_ gas detectors. Future work should focus on optimizing sensor performance, particularly response and recovery kinetics, and evaluating the long-term stability and selectivity in diverse gaseous atmospheres. The successful fabrication of these high-quality nanocrystalline perovskite films demonstrates that PED is a robust and precisely controllable method, suggesting its broader applicability for depositing other functional oxide materials.

## Figures and Tables

**Table 1:** Pulsed Electron Deposition process parameters for the production of La(Sr)CoO_3_ thin films.

Process Parameter	Value
Pulse Electron Energy, E (kV)	10
Gap, [mm]	2.5–3
Target–substrate, d (mm)	50–53
Oxygen partial pressure, p_O2_ (Pa)	0.973; (7.3–7.6 × 10^−3^ Torr)
Substrate temperature, T_s_ (°C) (radiative heater)	~750
Repetition rate, f (Hz)	1–3 Hz (predeposition) 10 Hz (process)
Number of shots	50,000 (predeposition) 200,000 (process)
Pulse deposition ratio (Å/pulse)	1–3.5 × 10^−3^
C monitor	135–145
Substrates	MgO [001] Si [001]

**Table 2:** Results of EDS chemical composition analyses of micro-area La(Sr)CoO_3_ thin films. Correction method: PhiRhoZ, Type: OxyByDiff-oxygen content was estimated using stoichiometry.

Thin Film	La				Sr				Co				O			
	Thin Film		Droplets, Particles		Thin Film		Droplets, Particles		Thin Film		Droplets, Particles		Thin Film		Droplets, Particles	
	wt.%	at.%	wt.%	at.%	wt.%	at.%	wt.%	at.%	wt.%	at.%	wt.%	at.%	wt.%	at.%	wt.%	at.%
LaCoO_3_ (stech.)	56.5	20	-	-	-	-	-	-	24	20	-	-	19.5	60	-	-
LaCoO_3_	55.9	18.2	58.7	21.0	-	-	-	-	24.2	19.1	21.7	18.2	19.9	62.7	19.6	60.8
La_0.9_Sr_0.1_CoO_3_	51.2	18.7	55.4	18.1	3.9	2.5	5.3	4.7	24.5	20.1	27.3	23.7	20.4	58.7	12.0	53.5
La_0.8_Sr_0.2_CoO_3_	47.3	16.2	50.0	12.1	7.5	4.1	11.0	7.9	25.2	22.3	28.3	25.5	20.0	57.4	10.7	54.5

**Table 3:** Roughness parameters of La(Sr)CoO_3_ thin films.

Sample/Thin Film	Roughness Parameter, (nm)		
	Rq	Ra	Rmax
LaCoO_3_	11.1	8.9	73.5
La_0.9_Co_0.1_O_3_	4.1	3.2	29.4
La_0.8_Co_0.2_O_3_	7.6	5.6	61.4

**Table 4:** La(Sr)CoO_3_ thin film NO_2_-sensing response (Resp), sensitivity (S), response (t_res_) and recovery (t_rec_) times measured for 250–400 °C.

Thin Film	Temperature T [°C]	NO_2_ Sensing Response, Resp	Thin Film Sensitivity to NO_2_, S [%]	Response Time t_res_, [min]	Recovery Time t_rec_, [min]
LaCoO_3_	250	1.1	6.2	31	30
	300	1.0	4.3	32	30
	350	1.0	1.4	30	30
	400	1.0	0.4	30	31
La_0.9_Sr_0.1_CoO_3_	250	1.1	6.6	29	29
	300	1.1	4.9	28	28
	350	1.0	1.9	29	30
	400	1.0	0.3	29	28
La_0.8_Sr_0.2_CoO_3_	250	1.1	8.3	25	23
	300	1.1	5.3	26	21
	350	1.0	2.5	24	21
	400	1.0	0.1	23	22

## Data Availability

The original contributions presented in this study are included in the article. Further inquiries can be directed to the corresponding author.
